# The Distribution of eIF4E-Family Members across Insecta

**DOI:** 10.1155/2012/960420

**Published:** 2012-06-13

**Authors:** Gritta Tettweiler, Michelle Kowanda, Paul Lasko, Nahum Sonenberg, Greco Hernández

**Affiliations:** ^1^Department of Biology, McGill University, 1205 Dr. Penfield, Montreal, QC, Canada H3A 1B1; ^2^Department of Biochemistry and Goodman Cancer Research Center, McGill University, Montreal, QC, Canada H3A 1A3; ^3^Division of Basic Research, National Institute for Cancer (INCan), Avenida San Fernando No. 22, Tlalpan, 14080 Mexico City, DF, Mexico

## Abstract

Insects are part of the earliest faunas that invaded terrestrial environments and are the first organisms that evolved controlled flight. Nowadays, insects are the most diverse animal group on the planet and comprise the majority of extant animal species described. Moreover, they have a huge impact in the biosphere as well as in all aspects of human life and economy; therefore understanding all aspects of insect biology is of great importance. In insects, as in all cells, translation is a fundamental process for gene expression. However, translation in insects has been mostly studied only in the model organism *Drosophila melanogaster*. We used all publicly available genomic sequences to investigate in insects the distribution of the genes encoding the cap-binding protein *eIF4E*, a protein that plays a crucial role in eukaryotic translation. We found that there is a diversity of multiple ortholog genes encoding eIF4E isoforms within the genus *Drosophila*. In striking contrast, insects outside this genus contain only a single *eIF4E* gene, related to *D. melanogaster* eIF4E-1. We also found that all insect species here analyzed contain only one Class II gene, termed *4E-HP*. We discuss the possible evolutionary causes originating the multiplicity of *eIF4E* genes within the genus *Drosophila*.

## 1. Introduction

Insects are the most diverse animal group on Earth and comprise over half of all extant described species, dominating thus all terrestrial ecosystems [1–4]. Winged insects were the first organisms that evolved controlled flight, some 120, 200, and 300 million years (Myr) before flying reptiles, birds, and bats, respectively. Indeed, wings are believed to have led largely to the spectacular diversification of insects because they were able to explore and invade all terrestrial ecosystems, escape predators, and exploit scattered resources [2, 5]. Many studies show that insect diversity has been also strongly shaped by other evolutionary and ecological processes, including their relative ancient geological age, low extinction rate, ecological niches occupancy, sexual selection, and sexual conflict [1]. 

Insects originated 434–421 Myr ago during the Silurian Period, and it is suggested that earliest terrestrial faunas already included wingless insects [2, 5, 6]. Indeed, the aquatic-terrestrial transition of insect ancestors is associated with the earliest vascular land plants fossils. Thus, it is thought that true insects evolved from an aquatic arthropod that formed an ecological association with the earliest vascular plants and subsequently both lineages coevolved [2, 6]. By the Permian (299–251 Myr ago) nearly all extant insect orders already have emerged, and later a second spectacular radiation happened in the Jurassic. Insects have been diverging ever since [2, 7, 8]. Winged insects, which account for more than 98% of the class Insecta, emerged when early arborescent plants evolved (pteridophytes, mostly ferns, and horsetails) 380–354 Myr ago (during the Devonian). It is hypothesized that insect flight arose as an adaptation to the increasing height of trees, and that a number of highly successful insect species coevolved with flowering plants [2, 5, 6, 9].

Besides their crucial ecological importance in all terrestrial ecosystems, insects have a huge direct impact in all aspects of human life and economy. In agriculture, some species cause huge damage to crops (e.g., aphids and weevil beetles), whilst others are of great benefit to flowering plants, which depend on pollinating species (e.g., bees, wasp, and butterflies). There are many species that can spread human pathogens (e.g., mosquitoes, fleas, and bed bugs) as well as key model organisms for basic research (*Drosophila*). Furthermore, several species serve as research objects for social behavior studies (e.g., bees and ants). Because of their overall significance, for many years immense efforts have been put forward to studying all aspects of insect biology. However, many biological processes, including translation, are still poorly studied at the molecular level. Therefore, further characterization of insect translation is necessary.

Most eukaryotic mRNAs are translated by a cap-dependent mechanism, whereby the mRNA is recruited to the ribosome through recognition of the 5′ cap structure (m^7^GpppN, where N is any nucleotide) by the cap-binding protein eIF4E in complex with the scaffold protein eIF4G and the RNA helicase eIF4A [10, 11]. Three-dimensional studies demonstrated that eIF4E associated to cap-analogues resembles “cupped-hands” in which the cap structure is stacked between two highly conserved tryptophan residues (Trp-56 and Trp-102 of mouse eIF4E) through *π* bond interactions. A third conserved tryptophan residue (Trp-166 of mouse eIF4E) binds the N^7^-methyl moiety of the cap structure [12–15]. Due to its pivotal role in translation, eIF4E activity is tightly regulated. Perhaps the most prominent regulatory mechanism is performed by eIF4E-binding proteins (4E-BPs), which bind eIF4E via an eIF4E-binding motif that is shared with eIF4G. 4E-BPs act as competitive inhibitors of eIF4E-eIF4G interaction and therefore of translation [10, 16, 17]. Another mechanism regulating eIF4E activity in some metazoans, including human, *Drosophila*, and *Aplysia*, is by phosphorylation of Ser-209 (mouse protein numbering; Ser251 in *Drosophila* eIF4E-1) [18–20].

Among insects, the unique translation initiation machinery that has been studied thus far is that from *D. melanogaster*. This species possesses seven genes encoding eight eIF4E cognates, one of them being 4E-HP (eIF4E-homolog protein) [19, 21–27]. All residues involved in 5′ cap structure binding are conserved in all eIF4Es [22–26], and experimental evidence confirmed their ability to bind this structure [21, 24, 26]. Likewise, most residues involved in eIF4G and 4E-BP binding are conserved and yeast two-hybrid experiments showed that all of them, except for eIF4E-6 and 4E-HP, interact with both proteins [26]. A functional assay showed that *D. melanogaster* eIF4E-1, eIF4E-2, eIF4E-4, eIF4E-5, and eIF4E-7, but not eIF4E-3 and eIF4E-6, are able to phenotypically rescue a lethal *eIF4E*-deficient yeast strain [26]. eIF4E-1 loss-of-function mutations cause growth arrest, severe embryonic defects, and lead to embryonic lethality [19, 28–30], and phosphorylation of eIF4E-1 at Ser251 is necessary for growth of the whole organisms [19]. Evidence supports the idea that there is physiological specialization of eIF4E cognates. While global translation is performed by eIF4E-1 [19, 28], eIF4E-3 is a testis-specific factor promoting translation in this tissue [31], eIF4E-5 might be involved in autophagy [32] and 4E-HP is a translational repressor [27, 33, 34]. Moreover, other activities have been reported for eIF4E-1, including a role in neurogenesis [35, 36] and a nuclear role in splicing [37]. Interactions of eIF4E-1 with different proteins, including 4E-BP [26, 38], Cup [39], Diap1 [40], and Ago2-Risc complex [41], have been described. Additionally, 4E-HP was found to interact with the RNA helicase Belle [42].

Recent advances in sequencing technology allow comparative analysis of multiple genomes across a wide range of evolutionarily related species. Thus, gene and protein annotation of twelve different *Drosophila* species [43] and from other insect species [44, 45] are now available. Here we investigated the distribution of the cap-binding proteins eIF4E and 4E-HP across the class Insecta.

## 2. Material and Methods

We compared annotated protein sequences of insects eIF4E-family members obtained from all publicly accessible databases, that is, http://umbicc3-215.umbi.umd.edu/ [45] and from several sequencing projects available in the NCBI GenBank NR, http://flybase.org/ and in http://www.butterflybase.org/ [44]. The genomes analyzed were from 12 *Drosophila* species [43], *Aedes aegypti*, *Anopheles gambiae* (all Diptera), *Camponotus floridanus, Harpegnathos saltator, Apis mellifera, Nasonia vitripennis *(all Hymenoptera), *Tribolium castaneum* (Coleoptera), *Manduca sexta*, *Spodoptera frugiperda*, *Heliconius melpomene*, *Bombix mori*, *Papilio xuthus* (all Lepidoptera), and *Acyrthosiphon pisum* (Hemiptera).  Table 1 shows all annotated genes and the proteins they encode that were analyzed in this study. Incomplete sequences and sequences encoding partial putative proteins were excluded. Amino acid sequences were aligned using ClustalW [46, 47] with the Biology Workbench bioinformatics package and improved by eye. Phylograms were assembled by neighbor-joining using *MEGA5* program [48].

Jagus and colleagues proposed a classification of eIF4Es from 230 species into three classes according to variations in the residues Trp-43 and Trp-56 (human eIF4E numbering) [45, 49]. Class I members contain both Trp residues; Class II members contain Tyr, Phe, or Leu at the first position and Tyr or Phe at the second position; Class III proteins contain Trp at the first position and Cys or Tyr at the second position [45, 49]. In the present study we will follow this classification. Since *D. melanogaster* is one of the most characterized model organisms and thus the best-studied species of all insects (whose entire genome is available for over a decade now (http://flybase.org/ [50]), and because among insects only eIF4Es and 4E-HP from *D. melanogaster* have been characterized [19, 21–42], we chose *D. melanogaster* eIF4Es sequences, numbering and nomenclature (http://flybase.org/ [25, 26]) as a reference. To avoid misunderstanding with another nomenclature [45, 49], here we will keep the fly database (http://flybase.org/) nomenclature, referring when necessary, to the Class each eIF4E belongs to.

## 3. Results and Discussion

### 3.1. eIF4E Proteins across the Genus Drosophila

**Figure 1:**
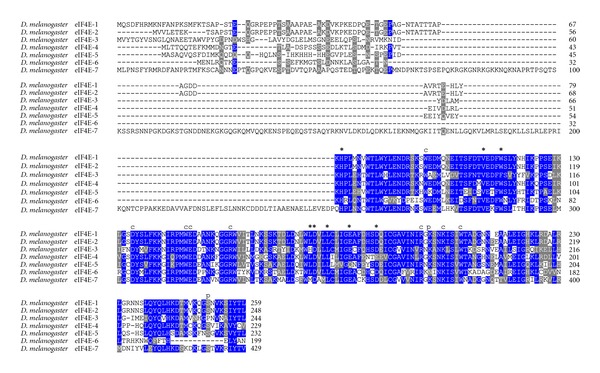
Seven eIF4E cognates in *D. melanogaster*. (A) ClustalW alignment of amino acid sequences representing Class I eIF4E family members from *D. melanogaster*. An extended version of eIF4E-6, not detected in previous studies, is included. Identical (blue) or conservative (gray) amino acid residues in at least 70% of sequences are highlighted. Conservative substitutions groups are STA, or NEQK, or NHQK, or NDEQ, or QHRK, or MILV, or MILF, or HY, or FYW, or GA. Residues essential for eIF4G- and 4E-BP binding are marked ∗; residues involved in cap binding are marked lower case *c*; phosphorylatable Ser, as well as Lys described to form a salt bridge with P-Ser, are marked lower case *p*.

Gene duplication of eIF4E is particularly striking in *D. melanogaster* with seven different cognates of Class I eIF4Es (eIF4E-1 trough eIF4E-7) and one Class II gene, termed *4E-HP* [25, 26]. Although sequence comparisons of all *D. melanogaster* eIF4Es are shown elsewhere [25, 26], a comparison of these proteins including an extended version of eIF4E-6 (see below) is shown in Figure 1. Using BLAST searches, it became evident that gene duplication of eIF4E also happened across the entire genus *Drosophila*. Overall, 61 different Class I eIF4E-family members were identified in this genus. We found that *D. simulans*, *D. sechellia*, *D. erecta,* and *D. yakuba* contain each six *eIF4E* genes (*eIF4E-1*, -*3*, -*4*, -*5*, -*6*, and -*7*), *D. ananassae*, *D. willistoni,* and *D. virilis* contain each five (*eIF4E-1*, -*3*, -*4*, -*5*, and -*7*), *D. grimshawi*, *D. pseudoobscura,* and *D. persimilis* contain each four (*eIF4E-1*, *-3*, -*4*, and -*5*) and *D. mojavensis* contains three cognates (*eIF4E-4*, -*5*, and -*7*) (Table 1).

**Figure 2:**
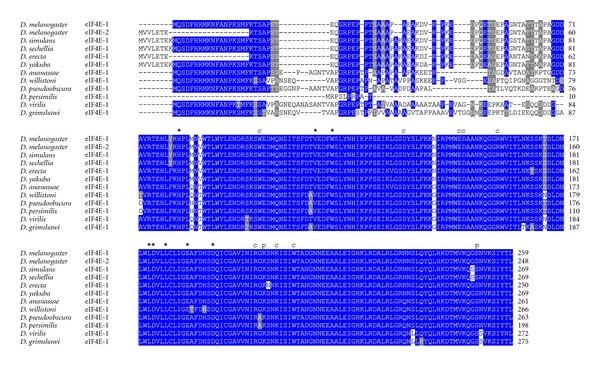
ClustalW alignment of amino acid sequences of eIF4E-1 orthologs from species of the genus *Drosophila*.

It has been shown that *D. melanogaster* eIF4E-1 and eIF4E-2 arise by alternative splicing from the same gene (*eIF4E-1/2*), both proteins differing only in amino acids in the N-terminus. While eIF4E-1 contains the peptide sequence MQSDFHRMKNFANPKSMF, eIF4E-2 contains MVVLETE instead [23, 24] (Figure 1). BLAST searches showed that the gene *eIF4E-1/2* exists only in *D. melanogaster*. In all other species this gene encodes only one protein, either eIF4E-1 (*D. ananassae*, *D. willistoni*, *D. pseudoobscura, D. virilis*, and *D. grimshawi)*, eIF4E-2 (*D. erecta*), or a protein with both N-termini fused (*D. simulans*, *D. sechellia,* and *D. yakuba)*. Interestingly, *D. persimilis* encodes an eIF4E-1 with a very short and divergent N-terminus (Figure 2). The high variability in eIF4E-1 N-terminus among *Drosophila* species suggests that this region of the protein has no biological relevance.

**Figure 3:**
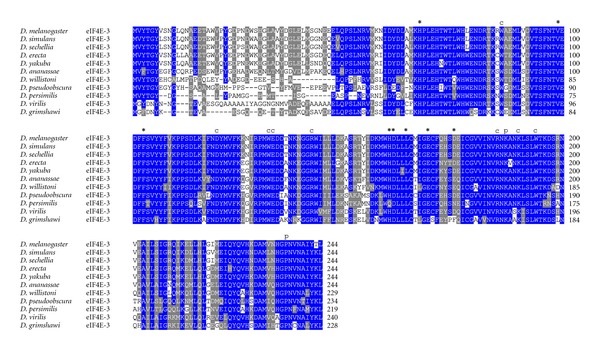
ClustalW alignment of amino acid sequences of eIF4E-3 orthologs from species of the genus *Drosophila*.

**Figure 4:**
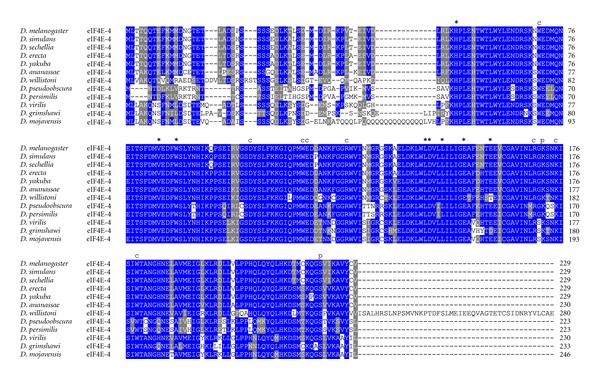
ClustalW alignment of amino acid sequences of eIF4E-4 orthologs from species of the genus *Drosophila*.

**Figure 5:**
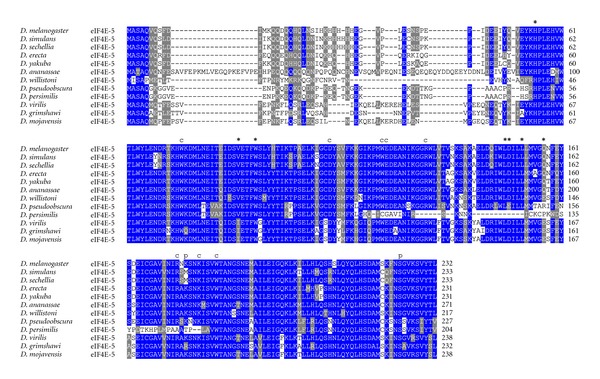
ClustalW alignment of amino acid sequences of eIF4E-5 orthologs from species of the genus *Drosophila*.

All residues involved in cap- and eIF4G/4E-BP-binding as well as for phosphorylation are conserved in eIF4E-1 from across the genus *Drosophila* (Figure 2). In eIF4E-3, residues involved in eIF4G/4E-BP binding are mutated in two positions, namely, Trp103>Phe, and Leu160>His (numbering according to *D. melanogaster* eIF4E-3; Figure 3). This significant alteration may explain the weak binding to eIF4G and 4E-BP shown in the yeast two-hybrid system [26]. Both changes are strongly conserved in eIF4E-3 across the genus *Drosophila*. Moreover, eIF4E-3 from all *Drosophila* species lack the counterpart of the phosphorylatable Ser251 of *D. melanogaster* eIF4E-1, possessing a proline instead [31] (Figure 3). *D. willistoni *eIF4E-4 displays the nonconservative amino acid exchange at position 160 Glu>Thr (numbering according to *D. melanogaster* eIF4E-4; Figure 4). eIF4E-5 varies considerably in length, ranging from 204 amino acids in *D. persimilis* to 271 amino acids in *D. ananassae*, and the N-terminus of eIF4E-5 (amino acids 1–53) is highly variable. However, eIF4E-5 is highly conserved from amino acid 54 on (Figure 5). *D. persimilis *eIF4E-5 also diverges from its orthologs in at least ten functionally important amino acids (Figure 5).

**Figure 6:**
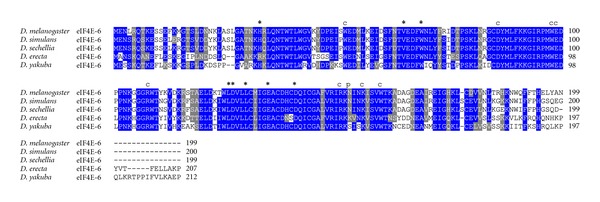
ClustalW alignment of amino acid sequences of eIF4E-6 orthologs from species of the genus *Drosophila*.

Recent experimental evidence supports an extended C-terminus of eIF4E-6 (Tettweiler, Hernández, Sonenberg, and Lasko, unpublished), not detected in previous studies [25, 26]. This extended eIF4E-6 showed the highest similarity to eIF4E-3 and has functionally important residues diverged from eIF4E-1 (Figure 1). One of the differences is a lack of phosphorylatable Ser251 (numbering of eIF4E-1). Surprisingly, although extended eIF4E-6 possesses all amino acids involved in eIF4G/4E-BP binding, experimental evidence showed that it does not bind either of them (Tettweiler, Hernández, Sonenberg, and Lasko, unpublished). The extended eIF4E-6 could only be detected in five species, all of which contain conserved residues important for cap binding (Figure 6). In *D. erecta*, a conserved substitution His>Arg is observed in position 33 (numbering according to *D. melanogaster* eIF4E-6; Figure 6), a residue essential for eIF4G/4E-BP binding. Similar to eIF4E-3, no eIF4E-6 from any species has the counterpart of eIF4E-1 Ser251 (Figure 6).

**Figure 7:**
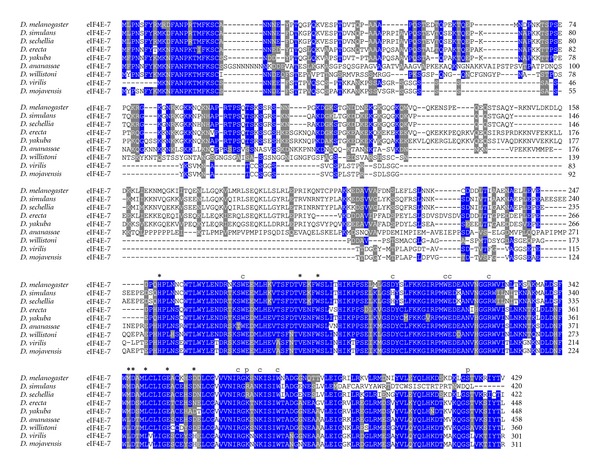
ClustalW alignment of amino acid sequences of eIF4E-7 orthologs from species of the genus *Drosophila*.

**Figure 8:**
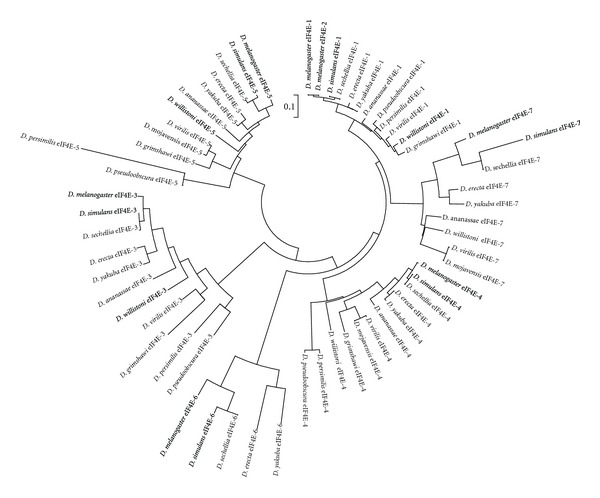
Radial phylogram computed from sequence alignments of eIF4Es from *Drosophila* species.

eIF4E-7 is the longest protein from Class I family members with 301 amino acids in *D. virilis* to 458 amino acids in *D. ananassae* (Figure 7). The high degree of discrepancy in length is attributed to the variability in the N-terminal moiety of the protein (Figure 7). Although eIF4E-7 orthologs are most similar to eIF4E-1, eIF4E-7 from all *Drosophila* species cluster together in separate phylogram branches (Figure 8). Several species are lacking functionally important residues in the eIF4E-7 C-terminus. In particular, in *D. simulans* eIF4E-7 the eIF4E-1 Ser251 counterpart is substituted by a Gln, albeit it is conserved in other *Drosophila* species (Figure 7).

Overall, our analyses indicate that the seven eIF4E-cognates in the genus *Drosophila* form discrete clusters (Figure 8), indicating that they evolved separately from each other before the radiation of ancestral *Drosophila* into the current species.

### 3.2. eIF4E Proteins in Other Insects

**Figure 9:**
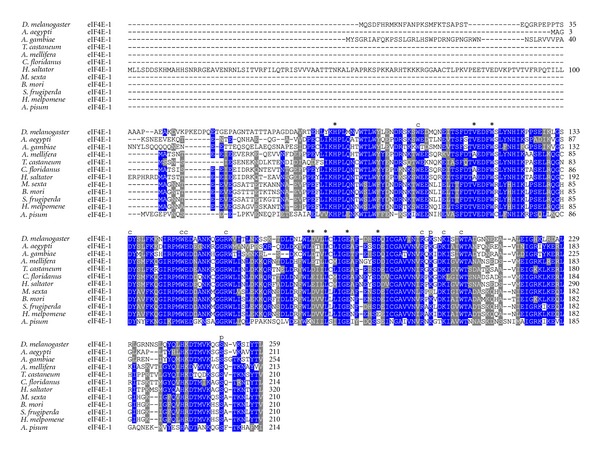
Orthologs of *D. melanogaster* eIF4E-1 in other insects. ClustalW alignment of amino acid sequences of eIF4E-1 orthologs from diverse insect species.

**Figure 10:**
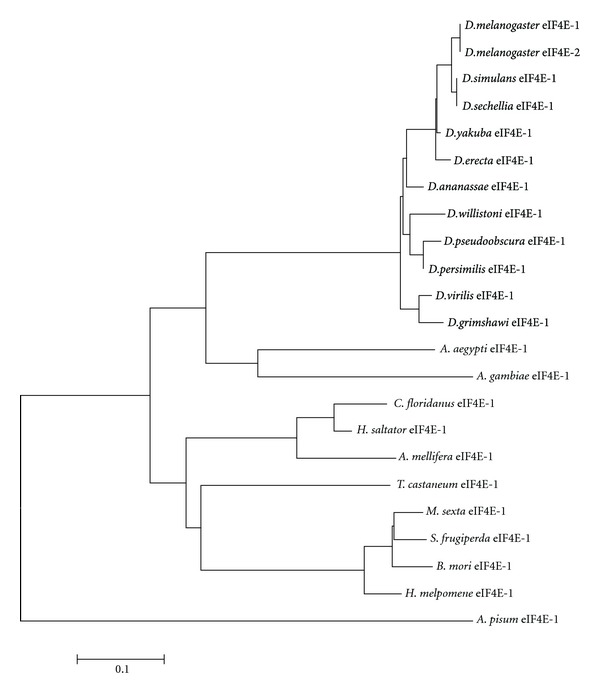
Phylogram computed from sequence alignments of eIF4E-1 from diverse insect species.

We analyzed protein annotations from all insect genomes that are publicly available. These include species representing non-*Drosophila* Diptera, as well as Hymenoptera, Coleoptera, Lepidoptera, and Hemiptera. Outside of the genus *Drosophila*, eleven more Class I eIF4Es were identified in different insect species (Figures 9 and 10). In contrast to *Drosophila* species, which contain three to seven different Class I eIF4Es cognates, we identified only a single Class I *eIF4E* gene in each insect genome analyzed, all of them related to *D. melanogaster* eIF4E-1 and with a highly variable N-terminus moiety (Figure 9). All amino acids described to be involved in cap and eIF4G/4E-BP binding are conserved in all insect eIF4Es analyzed. The exception is Leu174 (numbering according to *D. melanogaster* eIF4E-1), which is exchanged to Lys in *A. pisum* eIF4E-1.

Several evolutionary forces could account for the multiplicity of eIF4E genes in *Drosophila* genus, as opposed to the other insect lineages containing only one *eIF4E* gene. Diptera experienced three episodes of explosive radiation, one of them happened during the emergence of Schizophora (close relatives of *D. melanogaster*) in the early Tertiary Period (65 MYA). The Schizophora radiation originated most of the family-level diversity in Diptera, accounting for more than a third of extant fly diversity [2, 51–53]. Interestingly, the temporal pattern of fruit flies speciation corresponds with the major periods of climate cooling and habitat fragmentation during the Cenozoic Era, which could be one of the causes for stimulating the rapid fruit flies speciation [52]. The vigorous burst of diversification of the Schizophora was also coincident with the emergence of some developmental novelties, including the ptilinal sac, an improved escape mechanism for the fly from its puparium [53]. Since flies originated in wet environments, it has been suggested that the emergence of an impervious pupation to their surrounding allowed flies to adapt to almost all substrates and to occupy a broad range of trophic niches [53]. The explosive diversification of schizophoran could have induced the repeated events of *eIF4E* duplication in *Drosophila* species. It is conceivable that specific modes of temporal and spatial regulation of protein synthesis driven by different eIF4E isoforms conferred an adaptive advantage to these environmental changes.

At the molecular level, genomic studies revealed that repeated tandem gene duplication has generated ~80% of the nascent genes during the *D. melanogaster* subgroup evolution, and that retroposition has generated ~10% of the new genes in these species [54, 55]. Five to eleven new functional genes per million years were originated during evolution of this lineage [54, 55]. These findings may explain that *D. melanogaster eIF4E-1/2*, *eIF4E-3*, *eIF4E-4,* and *eIF4E-5* genes lie within a narrow region of the chromosome 3L and share exon/intron genomic structure [23, 24, 26]. Thus, it is conceivable that these genes originated by tandem duplication of an original *eIF4E-1* gene. On the other hand, *eIF4E-6* and *eIF4E-7* genes, which lie in different chromosomes and contain no introns in the core region of the genes [26], could have originated by retroposition events from *eIF4E-3* and *eIF4E-1*, respectively. Noteworthy, *D. mojavensis* only encodes eIF4E-4, -5, and -7, but not eIF4E-1. Since eIF4E-7 appears to be an extended eIF4E-1, we speculate that eIF4E-7 functions for eIF4E-1 in this species, which at a certain point of evolution lost the original *eIF4E-1* gene. When available in the near future, the chromosomic location of *D. mojavensis eIF4E-7* gene could corroborate this hypothesis.

### 3.3. 4E-HP in the Genus Drosophila

**Figure 11:**
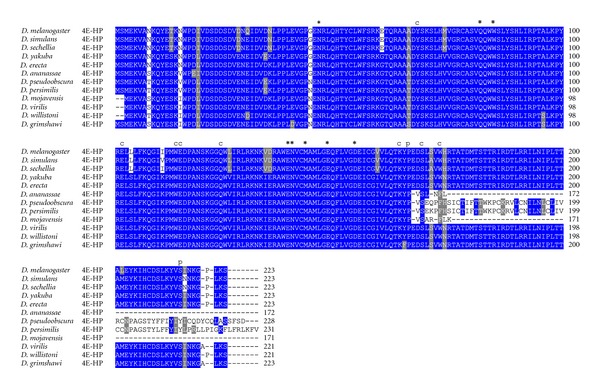
ClustalW alignment of amino acid sequences of 4E-HP orthologs from species of the genus *Drosophila*.

We also analyzed Class II eIF4E, namely, 4E-HP, in species of the genus *Drosophila*. In a striking contrast to all eIF4Es, a single copy of the *4E-HP* gene was identified in each *Drosophila* species. Interestingly, 4E-HP displays an unusually strong conservation in the N-terminal moiety of the protein and residues important for eIF4G/4E-BP binding diverge considerably from eIF4E-1 in all *Drosophila* species (Figure 11). This is the case of Asn46, Gln82, Glu139, Asn140, and Met143 (positions refer to *D. melanogaster* 4E-HP), which are His, Glu, Leu, Asp, and Leu residues in most *D. melanogaster* eIF4Es, respectively. Accordingly, *D. melanogaster *4E-HP does not bind eIF4G [26] but it interacts with Bicoid (bcd) and Brain Tumor (Brat) instead [27, 33]. Many residues critical for cap binding underwent both conservative and nonconservative mutations in 4E-HP from all *Drosophila* species analyzed. Thus, Tyr68, Glu102, Gln124, Lys164, Pro166, and Ser169 are Trp, Asp, Arg, Arg, Lys, and Lys in all *D. melanogaster* eIF4Es, respectively. The counterpart of phosphorylatable Ser251 in *D. melanogaster* eIF4E-1 is conserved in most species of the genus *Drosophila*. Finally, *D. ananassae* and *D. mojavensis* 4E-HP is considerably shorter than 4E-HP in other species (Figure 11).

### 3.4. 4E-HP in Other Insects

**Figure 12:**
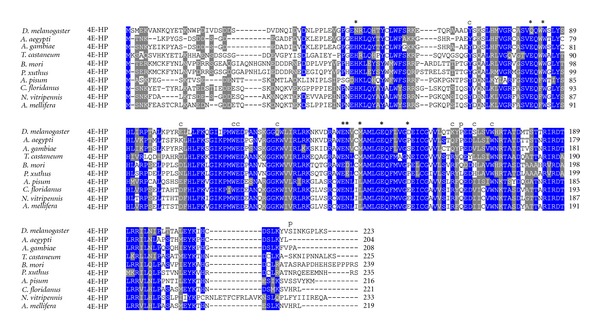
Orthologs of *D. melanogaster* 4E-HP in diverse insect species. ClustalW alignment of amino acid sequences of 4E-HP orthologs from diverse insect species.

**Figure 13:**
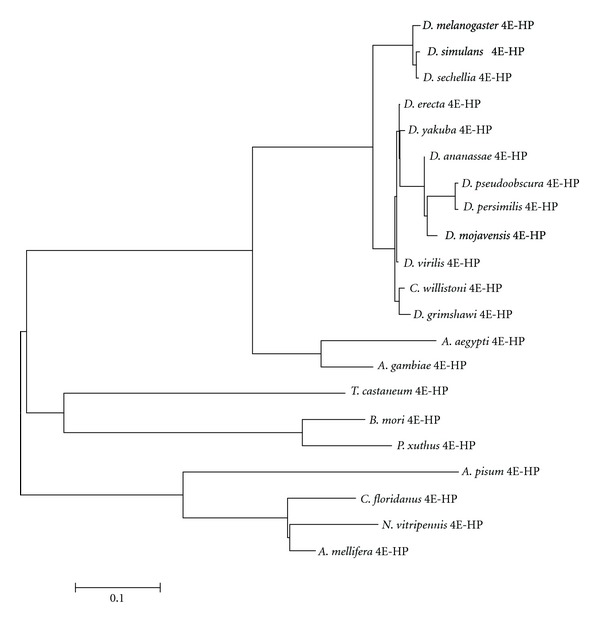
Phylogram computed from sequence alignments of 4E-HP from diverse insect species.

Further BLAST searches identified again single-copy *4E-HP* genes in other insect species. Sequence comparison showed a strong conservation in the core region of the protein, albeit N- and C-terminus are less conserved (Figure 12). In contrast to 4E-HP from *Drosophila* species, all residues important for eIF4G/4E-BP binding in eIF4Es are conserved in 4E-HP from all analyzed insects outside the genus *Drosophila*. This might suggest that 4E-HP in non-*Drosophila* insects do bind eIF4G/4E-BP. Similar to 4E-HP from all *Drosophila* species, most residues critical for cap-binding also show conservative changes in 4E-HP from Insecta species. The counterpart of phosphorylatable Ser251 of eIF4E-1 is only conserved in 4E-HP from *D. melanogaster*, *T. castaneum,* and *A. pisum* (Figure 12). A phylogram showing the relationships among 4E-HPs from all insects analyzed is shown in Figure 13.

Phylograms construction including all *Drosophila* 4E-HP and eIF4E sequences showed that all 4E-HPs cluster separately from all eIF4Es (not shown). Moreover, 4E-HP is widespread across metazoa, plants, and some fungi [45], and the *D. melanogaster *and human 4E-HP are able to bind the 5′ cap structure of the mRNA but not eIF4G [26, 56], thereby acting as a translational repressor of mRNAs associated to 4E-HP [27, 33, 34]. This, together with the findings that the *A. thaliana* [57] (termed nCBP) and the *C. elegans* [58] (termed IFE-4) orthologs can compete with reticulocyte eIF4E to reduce m^7^GTP binding and can be found associated with small ribosomal subunits, respectively, which is consistent with a regulatory function, led to the suggestion that 4E-HP diverged from a widespread ancestral Class I eIF4E into a translational repressor in mammals and in *Drosophila* [59]. This is supported by the observation that all residues important for eIF4G/4E-BP binding in eIF4Es are highly conserved in 4E-HP from non-*Drosophila* insects, but not in *Drosophila* species (Figure 12). Thus, 4E-HP from insects outside the genus *Drosophila* should bind eIF4G and promote translation. It is important to experimentally analyze this controversial hypothesis.

### 3.5. Class III eIF4Es

Among insects, only two partial Class III eIF4Es were identified, one in *A. mellifera* and one in *H. coagulata*. Both are missing the start methionine and were therefore not further analyzed.

## 4. Concluding Remarks

Constant updating of genomic data and annotations as well as improved search algorithms provided a more comprehensive overview of insect eIF4E cognates than previously possible. Here we presented an updated analysis of eIF4Es and 4E-HP across Insecta. This analysis revealed an interesting observation, that is, that *eIF4E* is a single-copy gene in all insects analyzed, but in the genus *Drosophila* this gene underwent a striking multiplication along with the explosive radiation this lineage went through in the early Tertiary. eIF4E diversification led to variability of biochemical properties and physiological specialization, as documented for some *D. melanogaster *eIF4Es. It would be worthy to investigate whether this is also the case for other species with several eIF4E cognates, as sequence alignments showed how diverse this protein is in the genus *Drosophila.* It also would be interesting to search for novel, so far unknown, 4E-BPs in other *Drosophila* species. Moreover, it is possible that different eIF4Es could translate specific target mRNAs.

eIF4E from more insect species must be analyzed to obtain a better picture of the evolution and diversity of eIF4E in this group, and to see whether the rise of multiple *eIF4E* genes is found in other insect lineages too. If so, correlating eIF4E evolution with the natural history of those lineages might lead us to find general, underlying forces driving the translation apparatus evolution.

## Figures and Tables

**Table 1:** Overview of annotated genes analyzed in this study.

eIF4E paralogs within *D. melanogaster*.	Orthologs in other *Drosophila* species	Orthologs in other insects
*eIF4E-1/2 *(CG4035)	*D. simulans* GD12928	*A. aegypti* AAEL001916
	*D. sechellia* GM24878	*A. gambiae* AGAP007172
	*D. erecta* GG14044	*C. floridanus* EFN73765
	*D. yakuba* GE21247	*H. saltator* EFN83757
	*D. ananassae* GF23736	*A. mellifera* XP_624290.2
	*D. willistoni* GK20927	*T. castaneum* XP_973494
	*D. pseudoobscura* GA28658	*M. sexta* MSP00767
	*D. persimilis* GL12850	*S. frugiperda* AAK94897
	*D. virilis* GJ13832	*B. mori* BGIBMGA012674
	*D. grimshawi* GH16860	*H. melpomene* HMP00347
		*A. pisum* ACYPI001956

*eIF4E-3 *(CG8023)	*D. simulans* GD14067
	*D. sechellia* GM25034
	*D. erecta* GG14292
	*D. yakuba* GE20721
	*D. ananassae* GF25106
	*D. willistoni* GK17185
	*D. pseudoobscura* GA24628
	*D. persimilis* GL26506
	*D. virilis* GJ12520
	*D. grimshawi* GH15498

*eIF4E-4 *(CG10124)	*D. simulans* GD13118
	*D. sechellia* GM13832
	*D. erecta* GG15032
	*D. yakuba* GE20475
	*D. ananassae* GF10894
	*D. willistoni* GK12583
	*D. pseudoobscura* GA28599
	*D. persimilis* GL13241
	*D. virilis* GJ12668
	*D. grimshawi* GH15637
	*D. mojavensis* GI12684

*eIF4E-5 *(CG8277)	*D. simulans* GD14038
	*D. sechellia* GM25004
	*D. erecta* GG14453
	*D. yakuba* GE21642
	*D. ananassae* GF10327
	*D. willistoni* GK17737
	*D. pseudoobscura* GA28380
	*D. persimilis* GL18042
	*D. virilis* GJ13889
	*D. grimshawi* GH14978
	*D. mojavensis* GI13141

*eIF4E-6 *(CG1442)	*D. simulans* GD18002
	*D. sechellia* GM12271
	*D. erecta* GG12044
	*D. yakuba* GE10483
*eIF4E-7 *(CG32859)	*D. simulans* GD16435
	*D. sechellia* GM18997
	*D. erecta* GG12718
	*D. yakuba* GE16544
	*D. ananassae* GF22031
	*D. willistoni* GK16243
	*D. virilis* GJ16331
	*D. mojavensis* GI14982

*4E-HP *(CG33100)	*D. simulans* GD18325	*A. aegypti *AAEL005796
	*D. sechellia* GM23515	*A. gambiae *AGAP002948
	*D. erecta* GG12377	*T. castaneum* XP_970157
	*D. yakuba* GE10831	*B. mori* NP_001091833
	*D. ananassae* GF23230	*P. xuthus* BAG30778
	*D. willistoni* GK11937	*A. pisum *ACYPI000423
	*D. pseudoobscura* GA26519	*C. floridanus *EFN65857
	*D. persimilis* GL24147	*N. vitripennis *XP_003426016
	*D. virilis* GJ14537	*A. mellifera* XP_623570
	*D. grimshawi* GH19097	
	*D. mojavensis* GI23433	

## References

[1] Mayhew PJ (2007). Why are there so many insect species? Perspectives from fossils and phylogenies. *Biological Reviews*.

[2] Grimaldi DA, Engel MS (2005). *Evolution of the Insects*.

[3] Bisby FA, Roskov YR, Orrell TM, Nicolson D, Paglinawan LE (2010). Catalogue of life: 2010 annual checklist. *Species 2000*.

[4] Mora C, Titterson DP, Adl S, Simpson AGB, Worm B (2011). How many species are there on Earth and in the ocean. *PLoS Biology*.

[5] Engel MS, Grimaldi DA (2004). New light shed on the oldest insect. *Nature*.

[6] Gaunt MW, Miles MA (2002). An insect molecular clock dates the origin of the insects and accords with palaeontological and biogeographic landmarks. *Molecular Biology and Evolution*.

[7] Whitfield JB, Kjer KM (2008). Ancient rapid radiations of insects: challenges for phylogenetic analysis. *Annual Review of Entomology*.

[8] Jarzembowski EA, Ross AJ, Hart MB (1996). Insect origination and extinction in the Phanerozoic. *Biotic Recovery from Mass Extinction Events*.

[9] Kevan PG, Chaloner WG, Savile DBO (1975). Interrelationships of early terrestrial arthropods and plants. *Paleontology*.

[10] Sonenberg N, Hinnebusch AG (2009). Regulation of Translation Initiation in Eukaryotes: mechanisms and Biological Targets. *Cell*.

[11] Jackson RJ, Hellen CUT, Pestova TV (2010). The mechanism of eukaryotic translation initiation and principles of its regulation. *Nature Reviews Molecular Cell Biology*.

[12] Marcotrigiano J, Gingras AC, Sonenberg N, Burley SK (1997). Cocrystal structure of the messenger RNA 5’ cap-binding protein (elF4E) bound to 7-methyl-GDP. *Cell*.

[13] Matsuo H, Li H, McGuire AM (1997). Structure of translation factor elF4E bound to m7GDP and interaction with 4E-binding protein. *Nature Structural Biology*.

[14] Tomoo K, Shen X, Okabe K (2002). Crystal structures of 7-methylguanosine 5′-triphosphate (m7GTP)- and P1-7-methylguanosine-P3-adenosine-5′, 5′-triphosphate (m7GpppA)-bound human full-length eukaryotic initiation factor 4E: biological importance of the C-terminal flexible region. *Biochemical Journal*.

[15] Tomoo K, Shen X, Okabe K (2003). Structural features of human initiation factor 4E, studied by X-ray crystal analyses and molecular dynamics simulations. *Journal of Molecular Biology*.

[16] Richter JD, Sonenberg N (2005). Regulation of cap-dependent translation by eIF4E inhibitory proteins. *Nature*.

[17] Sonenberg N, Hinnebusch AG (2007). New modes of translational control in development, behavior, and disease. *Molecular Cell*.

[18] Furic L, Rong L, Larsson O (2010). EIF4E phosphorylation promotes tumorigenesis and is associated with prostate cancer progression. *Proceedings of the National Academy of Sciences of the United States of America*.

[19] Lachance PED, Miron M, Raught B, Sonenberg N, Lasko P (2002). Phosphorylation of eukaryotic translation initiation factor 4E is critical for growth. *Molecular and Cellular Biology*.

[20] Ross G, Dyer JR, Castellucci VF, Sossin WS (2006). Mnk is a negative regulator of cap-dependent translation in Aplysia neurons. *Journal of Neurochemistry*.

[21] Maroto FG, Sierra JM (1989). Purification and characterization of mRNA cap-binding protein from *Drosophila melanogaster* embryos. *Molecular and Cellular Biology*.

[22] Hernández G, Sierra JM (1995). Translation initiation factor eIF-4E from *Drosophila*: cDNA sequence and expression of the gene. *Biochimica et Biophysica Acta*.

[23] Hernández G, Diez Del Corral R, Santoyo J, Campuzano S, Sierra JM (1997). Localization, structure and expression of the gene for translation initiation factor eIF-4E from *Drosophila melanogaster*. *Molecular and General Genetics*.

[24] Lavoie CA, Lachance PED, Sonenberg N, Lasko P (1996). Alternatively spliced transcripts from the *Drosophila* eIF4E gene produce two different cap-binding proteins. *The Journal of Biological Chemistry*.

[25] Lasko P (2000). The *Drosophila melanogaster* genome: translation factors and RNA binding proteins. *Journal of Cell Biology*.

[26] Hernández G, Altmann M, Sierra JM (2005). Functional analysis of seven genes encoding eight translation initiation factor 4E (eIF4E) isoforms in *Drosophila*. *Mechanisms of Development*.

[27] Cho PF, Poulin F, Cho-Park YA (2005). A new paradigm for translational control: inhibition via 5′-3′ mRNA tethering by Bicoid and the eIF4E cognate 4EHP. *Cell*.

[28] Hernández G, Vázquez-Pianzola P, Sierra JM, Rivera-Pomar R (2004). Internal ribosome entry site drives cap-independent translation of reaper and heat shock protein 70 mRNAs in *Drosophila* embryos. *RNA*.

[29] McNeill H, Craig GM, Bateman JM (2008). Regulation of neurogenesis and epidermal growth factor receptor signaling by the insulin receptor/target of rapamycin pathway in *Drosophila*. *Genetics*.

[30] Gong L, Puri M, Ünlü M (2004). *Drosophila* ventral furrow morphogenesis: a proteomic analysis. *Development*.

[31] Hernández G, Han H, Gandin V, Ferreira T, Sonenberg N, Lasko P Drosophila eukaryotic initiation factor 4E-3 is essential in post-meiotic stages of spermatogenesis.

[32] Gorski SM, Chittaranjan S, Pleasance ED (2003). A SAGE approach to discovery of genes involved in autophagic cell death. *Current Biology*.

[33] Cho PF, Gamberi C, Cho-Park Y, Cho-Park IB, Lasko P, Sonenberg N (2006). Cap-dependent translational inhibition establishes two opposing morphogen gradients in *Drosophila* embryos. *Current Biology*.

[34] Villaescusa JC, Buratti C, Penkov D (2009). Cytoplasmic Prep1 interacts with 4EHP inhibiting Hoxb4 translation. *PLoS ONE*.

[35] Menon KP, Sanyal S, Habara Y (2004). The translational repressor Pumilio regulates presynaptic morphology and controls postsynaptic accumulation of translation factor eIF-4E. *Neuron*.

[36] Sigrist SJ, Thiel PR, Reiff DF, Lachance PED, Lasko P, Schuster CM (2000). Postsynaptic translation affects the efficacy and morphology of neuromuscular junctions. *Nature*.

[37] Graham PL, Yanowitz JL, Penn JKM, Deshpande G, Schedl P (2011). The translation initiation factor eif4e regulates the Sex-Specific expression of the master switch gene Sxl in *Drosophila melanogaster*. *PLoS Genetics*.

[38] Miron M, Verdú J, Lachance PED, Birnbaum MJ, Lasko PF, Sonenberg N (2001). The translational inhibitor 4E-BP is an effector of PI(3)K/Akt signalling and cell growth in *Drosophila*. *Nature Cell Biology*.

[39] Piccioni F, Zappavigna V, Verrotti AC (2005). A cup full of functions. *RNA Biology*.

[40] Lee SK, Lee JS, Shin KS, Yoo SJ (2007). Translation initiation factor 4E (eIF4E) is regulated by cell death inhibitor, Diap 1. *Molecules and Cells*.

[41] Iwasaki S, Kawamata T, Tomari Y (2009). *Drosophila* argonaute1 and argonaute2 employ distinct mechanisms for translational repression. *Molecular Cell*.

[42]  Yarunin A, Harris RE, Ashe MP, Ashe HL (2011). Patterning of the *Drosophila* oocyte by a sequential translation repression program involving the d4EHP and Belle translational repressors. *RNA Biology*.

[43] Clark AG, Eisen MB, Smith DR (2007). Evolution of genes and genomes on the *Drosophila* phylogeny. *Nature*.

[44] Papanicolaou A, Gebauer-Jung S, Blaxter ML, Owen McMillan W, Jiggins CD (2008). ButterflyBase: a platform for lepidopteran genomics. *Nucleic Acids Research*.

[45] Joshi B, Lee K, Maeder DL, Jagus R (2005). Phylogenetic analysis of eIF4E-family members. *BMC Evolutionary Biology*.

[46] Thompson JD, Higgins DG, Gibson TJ (1994). CLUSTAL W: improving the sensitivity of progressive multiple sequence alignment through sequence weighting, position-specific gap penalties and weight matrix choice. *Nucleic Acids Research*.

[47] Higgins DG, Thompson JD, Gibson TJ (1996). Using CLUSTAL for multiple sequence alignments. *Methods in Enzymology*.

[48] Tamura K (2011). MEGA5: molecular evolutionary genetics analysis using maximum likelihood, evolutionary distance, and maximum parsimony methods. *Molecular Biology and Evolution*.

[49] Rhoads RE, Dinkova TD, Jagus R (2007). Approaches for analyzing the differential activities and functions of eIF4E family members. *Methods in Enzymology*.

[50] Adams MD, Celniker SE, Holt RA (2000). The genome sequence of *Drosophila melanogaster*. *Science*.

[51] Yeates DK, Wiegmann BM, Courtney GW, Meier R, Lambkin C, Pape T (2007). Phylogeny and systematics of Diptera: two decades of progress and prospects. *Zootaxa*.

[52] Tamura K, Subramanian S, Kumar S (2004). Temporal patterns of fruit fly (*Drosophila*) evolution revealed by mutation clocks. *Molecular Biology and Evolution*.

[53] Wiegmann BM, Trautwein MD, Winkler IS (2011). Episodic radiations in the fly tree of life. *Proceedings of the National Academy of Sciences of the United States of America*.

[54] Zhou Q, Wang W (2008). On the origin and evolution of new genes-a genomic and experimental perspective. *Journal of Genetics and Genomics*.

[55] Zhou Q, Zhang G, Zhang Y (2008). On the origin of new genes in *Drosophila*. *Genome Research*.

[56] Rom E (1998). Cloning and characterization of 4E-HP, a novel mammalian eIF4E-related cap-binding protein. *The Journal of Biological Chemistry*.

[57] Ruud KA, Kuhlow C, Goss DJ, Browning KS (1998). Identification and characterization of a novel cap-binding protein from *Arabidopsis thaliana*. *The Journal of Biological Chemistry*.

[58] Dinkova TD, Keiper BD, Korneeva NL, Aamodt EJ, Rhoads RE (2005). Translation of a small subset of *Caenorhabditis elegans* mRNAs is dependent on a specific eukaryotic translation initiation factor 4E isoform. *Molecular and Cellular Biology*.

[59] Hernández G, Altmann M, Lasko P (2010). Origins and evolution of the mechanisms regulating translation initiation in eukaryotes. *Trends in Biochemical Sciences*.

